# Exploring digital narratives: A comprehensive dataset of household energy app reviews

**DOI:** 10.1016/j.dib.2023.109835

**Published:** 2023-11-19

**Authors:** Chandra Prakash Paneru, Cristian Toșa, Ari K.M. Tarigan

**Affiliations:** Department of Safety, Economics and Planning, University of Stavanger, Norway

**Keywords:** Sustainable energy, Smart innovation, Norway, Technology-driven behaviour change

## Abstract

This paper presents an anonymous dataset of 7999 user reviews covering five household energy mobile applications used in Norwegian households. Such reviews are usually available through the Google Play Store and Apple App Store platforms. They were collected using Python-based Google-Play-Scraper and App Store Scraper. To the best of our knowledge, this dataset represents a unique and valuable resource for investigating sustainable household energy behaviour within the specific context of Norway, where a considerable proportion of households already use these applications. Given the recent rise of mobile applications and the ongoing development of technological infrastructure worldwide, this dataset holds a potential for empirical research. It can provide valuable insights into daily energy practices, user sentiments, perceptions, and motivations for adopting digital solutions. Further, it can shed light on the potential of these solutions to drive sustainable behavioural change. Moreover, conducting the empirical analysis of this dataset can provide valuable insights to stakeholders involved in policy formulation, utility improvement, emissions reduction, and promotion of technology-driven behavioural change.

Specifications Table

**Table:** 

Subject	Renewable Energy, Sustainability and the Environment/ Business, Management and decision sciences
Specific subject area	The data article highlights the high relevance of mining user reviews of household energy apps for researchers, policymakers, utility companies, and smart digital solution developers.
Data format	Raw, Anonymised
Type of data	Table
Data collection	Data was collected by extracting publicly available reviews from the Google Play Store and Apple Appstore through an automated script. The Python program was used to execute the script.
Data source location	The dataset contains user reviews on five different household energy apps from Norway. Data and Python code were collected at the University of Stavanger, Norway and stored in the Open Science Framework (OSF) data repository.
Data accessibility	Repository name: Open Science Framework (OSF) Data identification number: 10.17605/OSF.IO/36YFP Direct link to data: http://doi.org/10.17605/OSF.IO/36YFP

## 1 Value of The Data

• The dataset consists of publicly available user reviews collected from Android and iOS platforms for five household energy applications available on the Norwegian market such as Tibber, Lyse, Fjordkraft, Elvidia, and True Energy.
• Although publicly available, data is scattered across different platforms and is not easily accessible in a consolidated format. Researchers save time and effort by not having to collect and collate this data. This dataset brings together 7999 subjective reviews provided by users of energy apps within Norwegian households, specifically related to the apps they utilise, making it a unique and valuable resource.
• Consumer-level energy data is challenging to gather, and researchers studying energy behaviour often struggle to obtain real data. This dataset, therefore, provides a much-needed resource for researchers, enabling them to undertake end-user energy behaviour studies.
• Given the empirical research gap on the efficacy of household energy apps in promoting sustainable household energy behaviours, this dataset can be used to study real-world insights into app users' attitudes, perceptions and intentions to adopt sustainable household energy practises (e.g., reducing energy consumption, improving energy efficiency, and aiding in demand response) as well as to understand the potential of such apps to enable future research on technology-driven behavioural change [1].
• The dataset enables a detailed case study of smart energy apps as an influential smart digital solution in the context of Norway, a country leading the digital transformation with a high level of digitalisation in its households [1,2]. The insights from the Norwegian context can provide valuable lessons for other contexts aiming for a digital energy transition.
• Moreover, this dataset enables researchers to perform text mining analyses, yielding valuable consumer insights (e.g., word clouds, content analysis, sentiment analysis, thematic analysis, topic modelling, etc.). These insights benefit researchers, policymakers, utility companies, and smart digital solution developers.
• Finally, the results of the analyses have the potential to guide the enhancement of the design, functionality, and user interface of these applications. Such enhancement can increase the likelihood of application adoption and improve household energy efficiency, thereby contributing to achieving the Sustainable Development Goals (SDGs).

## 2 Data Description

The research methodology of mining consumer reviews has gained popularity due to the expansion of the internet and the rising popularity of smart digital solutions across sectors [3,4]. The user-generated reviews provide a more user-focused perspective regarding different usage scenarios [5]. Although the reviews are inherently subjective, they represent a novel and valuable data source that can be easily acquired, less time-consuming, and potentially less susceptible to “social desirability biases” [6].

Through analysing feedback provided by the Norwegian Smart Energy app users [7], researchers can gain significant insights that may benefit researchers, utility companies, and policymakers worldwide who are engaged in transitioning to a digitalised energy system. The dataset offers an opportunity to conduct text-mining analyses revealing users' opinions, attitudes, and motivations towards sustainable energy practices. The analyses can shed light not only in terms of household energy usage patterns but also on their willingness to adopt smart digital solutions. This novel approach has the potential to contribute to policy formulation, improve public services, reduce emissions, and influence the way people use technology in relation to their energy-related choices.

The reviews are derived from five energy applications used in Norwegian households, sourced from the Google Play Store and Apple Appstore. The dataset includes reviews until August 7, 2023, providing a comprehensive record of reviews available on these platforms. The dataset shows different start dates for the reviews of each app, reflecting the phased release of the applications, as shown in Table 1.

**Table 1:** Starting date of the Reviews for each app and each platform.

Name of the App	Starting Date	
	Google Play	Appstore
Elvia	23.12.2011	01.01.2012
Fjordkraft	07.08.2015	29.07.2015
Lyse	22.05.2012	22.05.2012
Tibber	16.02.2017	24.10.2017
True Energy	16.02.2017	04.09.2021

Generally, these apps serve as smart digital tools for end-users to monitor and manage household energy consumption. They provide comprehensive energy insights, including electricity consumption patterns (hourly, daily, weekly, monthly, and yearly), spot energy prices, weather forecasts, and much more. Other energy information includes electricity bill information, energy-saving tips, reminders, and other relevant notifications. The level of energy feedback, visualisation and gamification elements varies from app to app.

The dataset is an Excel file containing 10 columns, each corresponding to a distinct dimension of user reviews. These attributes provide valuable contextual information, such as the user rating associated with each review. This well-structured dataset provides opportunities for comprehensive analysis, enabling a multidimensional exploration of users' perceptions and interactions with smart energy applications. This understanding provides researchers advantages and enables stakeholders to make well-informed decisions within the dynamic landscape of energy applications and digital energy solutions. Table 2 presents a brief description of each of the attributes related to each unique review I.D.

**Table 2:** Header of the raw dataset.

Attributes	Explanation
app_name	Name of the Smart Digital App
source	Review source (Google Play Store or App Store)
review_id	Unique review ID
review_date	Review date and timestamp
review_description_en	English translation of raw review data
rating	User rating (1–5)
review_title	Review title
developer_response	Developer response to user review
developer_response_date	Developer response date and timestamp
thumbs_up	Number of likes for the review

**Fig. 1:**
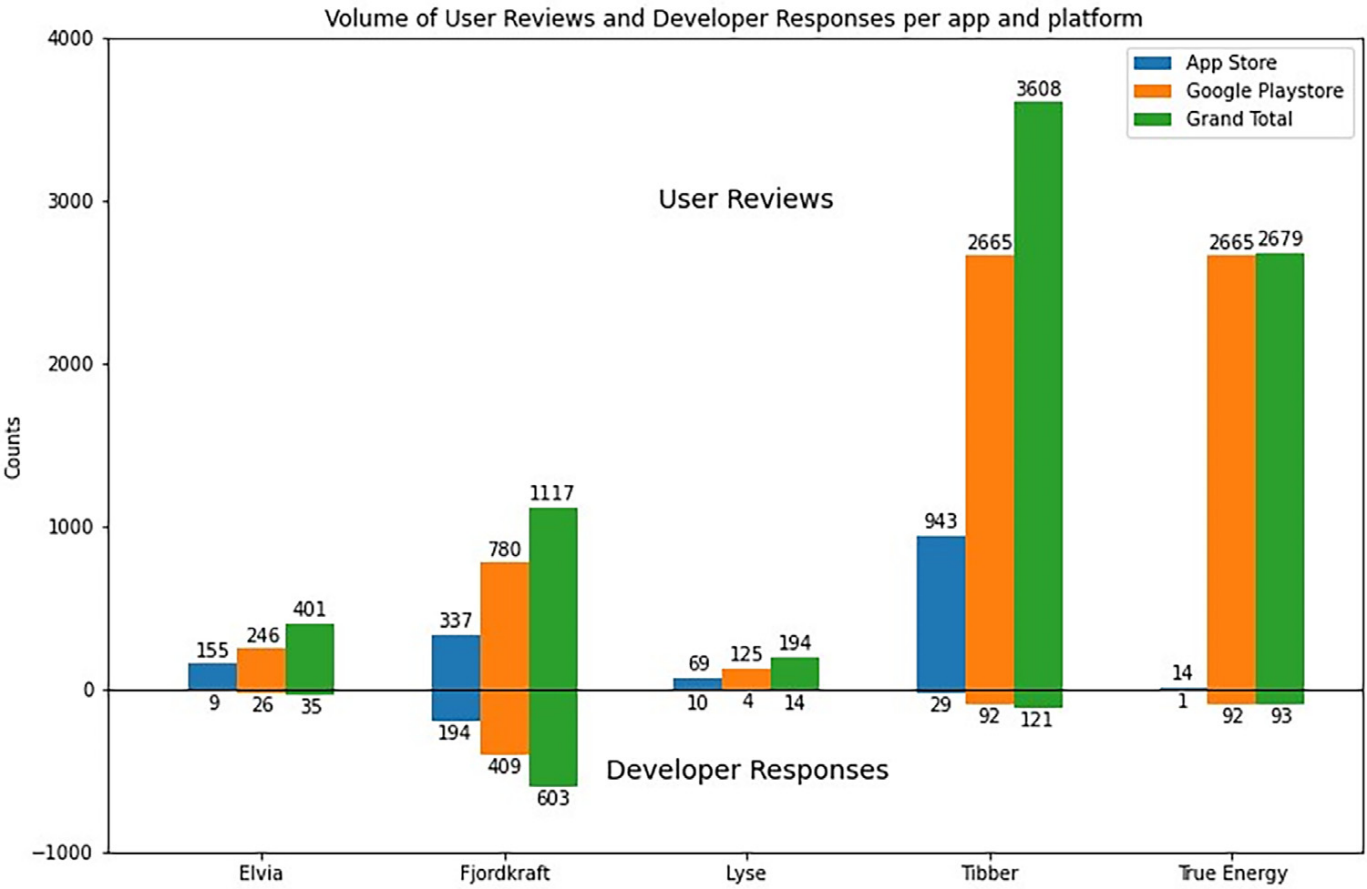
Distribution of user reviews and developer responses volume by app and platform.

**Fig. 2:**
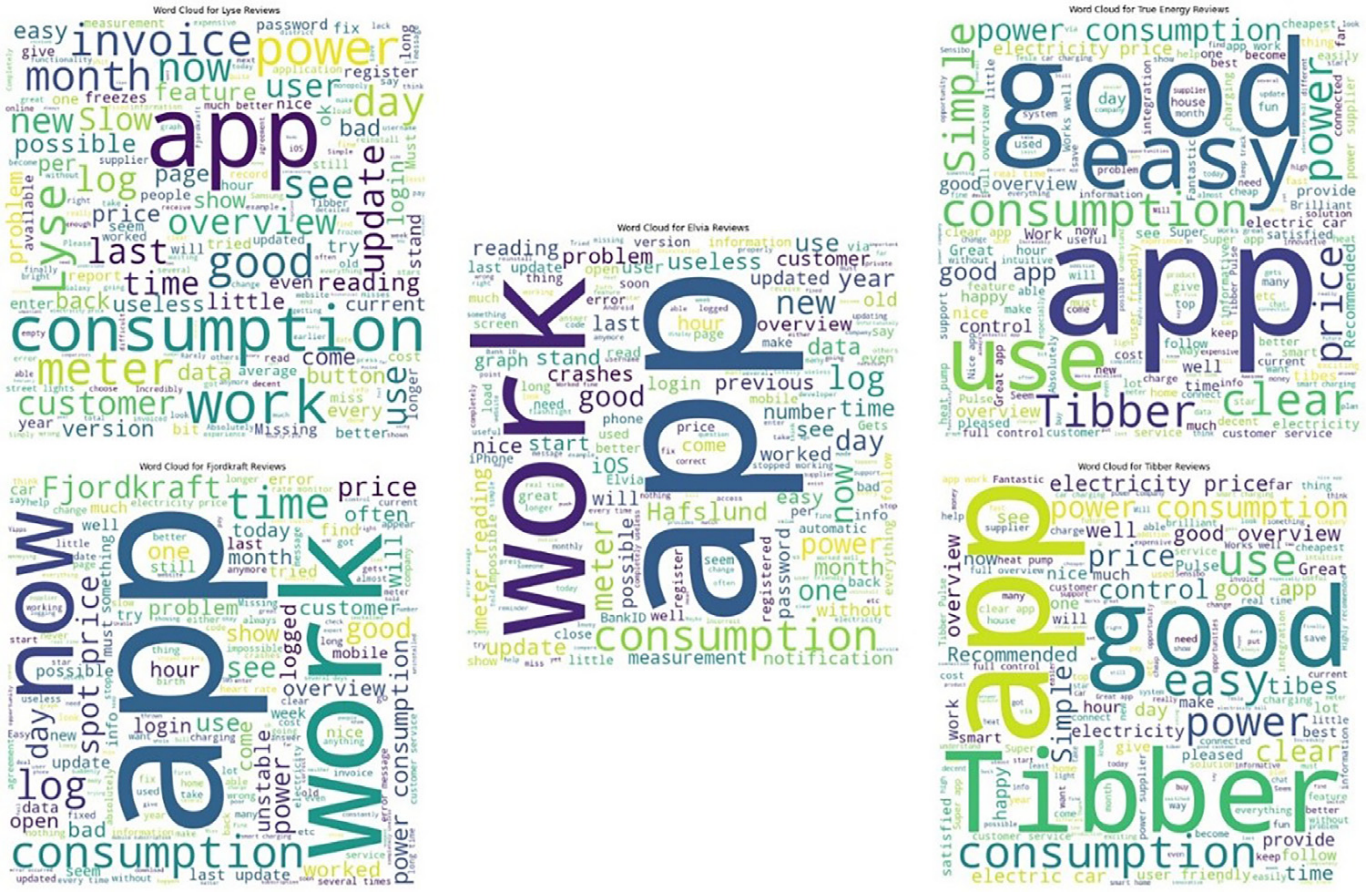
Word clouds on reviews for each app.

Fig. 1 shows the volume of user reviews and developer responses on each platform and each app. Fig. 2 is a word cloud for five apps, visualising the words that appear most frequently in the reviews for each app.

## 3 Experimental Design, Materials and Methods

The reviews from both the Google Play Store and Apple App Store were collected from the date the reviews were available on the platforms as shown in Table 1 and until 07.08.2023. The process and the Python scripts used for data scraping, merging, exporting, and analysing are provided in detail below to enable replicability of the method.

1. For data collection, the Python libraries “*google-play-scraper*” (available at https://pypi.org/project/google-play-scraper/) [8] and the “*app-store-scraper*” (available at https://pypi.org/project/app-store-scraper/) [9] were used [10]. The reviews were merged into a unified dataset and exported as a single Excel file. The Python file: “*full review scraper*” stored at this address: https://osf.io/mh3an [7] contains all the Python scripts with split-up scripts on each of these steps: scraping reviews for each app and platform, converting reviews to data frames for each app, merging the data frames and exporting the single dataset as an excel file.
2. The column labelled “*user_name*” was removed from the dataset to safeguard user identity and uphold privacy standards. The columns labelled “*review description*”, “*review title*”, and “*developer* response”, all containing unprocessed data in Norwegian language, were translated into English language using the Google Translate function in Google Sheets: (=GOOGLETRANSLATE(A1, “no”, “en”)). The translation function is applied to all entries for the abovementioned three columns. The column labelled “*thumbs_up*” remains unmodified. All remaining data in the dataset is presented in its unprocessed form.
3. The dataset contains 7999 reviews distributed across five apps and two app provision platforms, as itemised in Table 2 and is available as a unified Excel file at this address: https://osf.io/8qcsb [7]. This dataset can be used for further analysis or processing as the researcher requires.
4. Our preliminary data analysis included plotting the volume of user reviews and developer responses per app and platform and plotting word clouds for the reviews for each app.
5. For plotting the volume of user reviews and developer responses, the Python script is saved under the filename: “*reviews and responses -summary*” stored at this address: https://osf.io/sb8a7 [7] which guides through all the scripts and the steps followed: importing necessary libraries (Matplotlib and NumPy), organising data for reviews and responses, calculating the total for each app, creating bar plots for each category, setting axis limits and labels, adding numerical labels to the bars, creating a custom legend, setting the graph's title and textual labels, and finally displaying the plot.
6. The Python scripts used to process the user reviews to create textual data to generate word clouds are saved under the filename: “*wordcloud*” and stored at this address: https://osf.io/k5g2n. The script follows these steps: **a.** importing the necessary libraries (specifically pandas for data manipulation and the 'WordCloud' library for generating word clouds), **b.** reading the dataset from the unified Excel file containing user reviews, **c.** collecting and merging reviews into a single text string **d.** generating a word cloud for each app, **e.** Displaying word clouds using 'matplotlib.pyplot' library, and finally, **f.** saving the word clouds.
7. The Excel file, Python scripts and the plots can all be downloaded from the OSF data repository : http://doi.org/10.17605/OSF.IO/36YFP [7].

## Ethics Statement

This study was conducted in accordance with ethical guidelines and regulations. This study involved the analysis of publicly available user reviews. No informed consent was required because all the reviews were already publicly available. We ensured that sensitive user data was fully anonymised and no personal information was included in the dataset.

## CRediT authorship contribution statement

**Chandra Prakash Paneru:** Investigation, Methodology, Writing – original draft, Writing – review & editing. **Cristian Toșa:** Conceptualization, Writing – review & editing, Supervision. **Ari K.M. Tarigan:** Resources, Project administration, Supervision.

## Data Availability

A dataset of user reviews on Norwegian household energy apps (Original data) (OSF)
